# The Untold Story of the Caudal Skeleton in the Electric Eel (Ostariophysi: Gymnotiformes: *Electrophorus*)

**DOI:** 10.1371/journal.pone.0068719

**Published:** 2013-07-24

**Authors:** Carlos David de Santana, Richard P. Vari, Wolmar B. Wosiacki

**Affiliations:** 1 Division of Fishes, Department of Vertebrate Zoology, National Museum of Natural History, Smithsonian Institution, Washington, D. C., United States of America; 2 Setor de Ictiologia, Museu Paraense Emilio Goeldi, Belém, Pará, Brazil; Universität Bielefeld, Germany

## Abstract

Alternative hypotheses had been advanced as to the components forming the elongate fin coursing along the ventral margin of much of the body and tail from behind the abdominal region to the posterior margin of the tail in the Electric Eel, *Electrophorus electricus*. Although the original species description indicated that this fin was a composite of the caudal fin plus the elongate anal fin characteristic of other genera of the Gymnotiformes, subsequent researchers proposed that the posterior region of the fin was formed by the extension of the anal fin posteriorly to the tip of the tail, thereby forming a “false caudal fin.” Examination of ontogenetic series of the genus reveal that *Electrophorus* possesses a true caudal fin formed of a terminal centrum, hypural plate and a low number of caudal-fin rays. The confluence of the two fins is proposed as an additional autapomorphy for the genus. Under all alternative proposed hypotheses of relationships within the order Gymnotiformes, the presence of a caudal fin in *Electrophorus* optimized as being independent of the occurence of the morphologically equivalent structure in the Apteronotidae. Possible functional advantages to the presence of a caudal fin in the genus are discussed.

## Introduction

The order Gymnotiformes includes 33 genera and more than 200 extant species of Neotropical electric fishes plus one fossil form from the Late Miocene of Bolivia [1], [2]. Gymnotiforms inhabit freshwaters across the expanse from northern Argentina to southern Mexico in settings ranging from shallow, slow-flowing streams to deep rivers, with subsets of several families specialized for life in rapids and other high energy settings [3]–[6]. Species of gymnotiforms range in body size from miniatures of 50 mm total length such as *Hypopygus minissimus* [7] to the over 2.5 m total length of *Electrophorus electricus* [8]; a 50 times range notable in itself, but particularly striking in a lineage of circa only 200 species.

Arguably one of the most noteworthy characteristics of all gymnotiforms is their ability to produce electric organ discharges (EODs) which serve dual purposes - communication and exploration of the surrounding environment. Two alternative forms of such discharges occur among these electric fishes: pulse EODs (via myogenic organs) and wave EODs (via myogenic or neurogenic organs). *Electrophorus* is unique within the Gymnotiformes in having a third form of discharge of up to 600 volts used for hunting and self-defense [9], [10]. Such powerful discharges are dramatically apparent to anyone in contact with, or in close proximity, to these fishes in the water during a discharge. These shocks were reported by naturalists commencing early in the European exploration of the Neotropics, have been the subject of study by physiologists and are well known in popular lore [11].

*Electrophorus* was erected by Gill [12] to include the Electric Eel, *Gymnotus electricus* Linnaeus [13]. The description by Linneaus [13] was based on what was for the period a very detailed account and accompanying illustration by Gronovius [14] of a specimen probably originating in Suriname. Ichthyofaunal sampling over the following two and one-half centuries documented that *Electrophorus* has a broad distribution in low- and mid-elevation settings across the vast expanse encompassed by the Amazon and Orinoco basins and additionally through the river systems of northern Brazil and the Guianas between the mouths of those two major drainages [15], [16].

**Figure 1:**
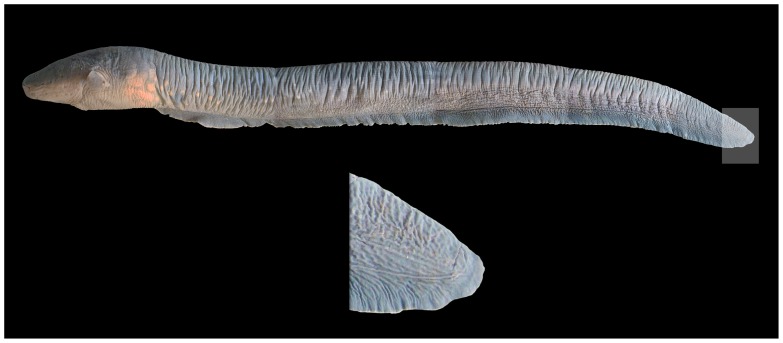
Lateral view of *Electrophorus electricus,* MPEG 25422, 1000 mm TL. Continuous compound fin along ventral surface of body and posteroventral portion of tail shown in upper figure and close up of compound anal-caudal fin at tip of tail in lower figure.

Various autapomorphies unique within the Ostariophysi distinguish *Electrophorus* [17], with one of the most prominent being the presence of three hypaxial electric organs (the Main, Hunter and Sach organs) versus a single hypaxial organ in adults of other gymnotiforms [1]. *Electrophorus* also has a highly vascularized oral respiratory organ with multiple folds that greatly increase its surface area [1], [9]; an elaboration unique to the genus among Neotropical electric fishes and critical for respiration in this obligatory air breather. The Electric Eel, moreover, differs from all other gymnotiforms in the elongate fin extending along the ventral surface of the body and tail from posterior of the abdominal cavity to the end of tail (Fig. 1; [18]). Other gymnotiforms conversely have the lengthy anal fin terminating further anteriorly along the tail.

Alternative hypotheses have been advanced concerning the components of the elongate fin coursing along the ventral surface of the body and tail of *Electrophorus.* Linnaeus [13] originally postulated that the anal fin of *Gymnotus electricus* (the *Electrophorus electricus* of this paper) was posteriorly continuous with the rays of the caudal-fin, i.e., the caudal fin is present. Subsequent authors ascribed to the alternative concept of the absence of a caudal fin in the genus. Intriguingly, the details of the unusual tail along the ventral and posterior margins of the body in *Electrophorus* have not been the subject of analysis to evaluate the two alternative hypotheses – that the fin at the posterior of the tail is a true caudal fin versus that the terminal portion of the elongate fin in the genus is a posterior extension of the anal fin to form a false caudal fin. We herein address that question and evaluate the results within the context of the divergent hypotheses of intraordinal phylogenetic relationships in the Gymnotiformes.

## Materials and Methods

Specimens were examined at, or borrowed from, the following institutions: AMNH, American Museum of Natural History, New York; AUM, Auburn University Museum, Auburn; ANSP, Academy of Natural Sciences of Drexel University, Philadelphia; FMNH, Field Museum of Natural History, Chicago; INHS, Illinois Natural History Survey, Champaign; KU, University of Kansas, Lawrence; MBUCV, Museo de Biologia de la Universidad Central de Venezuela, Caracas; MCZ, Museum of Comparative Zoology, Harvard University, Cambridge; MNRJ, Museu Nacional, Rio de Janeiro; MPEG, Museu Paraense Emílio Goeldi, Belém; MZUSP, Museu de Zoologia da Universidade de São Paulo, São Paulo; UF, Florida Museum of Natural History, Gainesville; NRM, Swedish Museum of Natural History, Stockholm; and USNM, National Museum of Natural History, Smithsonian Institution, Washington. The abbreviation TL in the text  = total length. Caudal fin skeletal morphology was assessed via radiographs and specimens cleared and counterstained for bone and cartilage following the procedure of Taylor & Van Dyke [19] (see List S1). Specimens with a damaged posterior region of the tail were excluded from the analysis. Due to the ontogenetically late onset of chondrification and calcification of the posterior portions of the body in the genus it was not feasible to provide informative photos of the caudal region in early life stages.

## Results and Discussion

### The Caudal Skeleton in *Electrophorus*

Well over two centuries ago, Linnaeus [13]: 427 commented that *Gymnotus electricus* had “*Pinna caudali obtufiffima anali annexa”* ( = the caudal fin very obtuse and joined to the anal). Information in that account indicated that his statement was most likely derived from a detailed description and illustration of a specimen of the species by Gronovius [14] rather than based on the examination of material of *G. electricus*. This concept of conjoined anal and caudal fins in what was later termed *Electrophorus electricus* (hereafter *Electrophorus*) then vanished without comment from the scientific literature for more than 200 years. The alternative accepted scenario was that the anal fin extended posteriorly to the end of tail in *Electrophorus* and formed what has been termed a false caudal fin [1], [17], [20]–[25]. An assumption that the terminal portion of the elongate fin in *Electrophorus* was a false, rather than true, caudal fin may have been, in part, based on the absence of the caudal fin in *Gymnotus,* a genus showing a number of derived characters with *Electrophorus*, with those two genera now forming the Gymnotidae. Comments as to a possible contrary arrangement were limited to remarks by Meunier & Kirschbaum [26], [27].

Meunier & Kirschbaum [26]: 216 briefly mentioned the possible presence of a caudal fin in *Electrophorus* as an alternative to the prevailing concept of an elongate anal fin extending posteriorly to the terminus of the tail. Soon thereafter Meunier & Kirschbaum [27]: 149 speculated again on the presence of a caudal skeleton in the genus, stating that “…the last vertebra is terminated by a small cartilage, which serves to support some lepidotrichia.” That observation notwithstanding, those authors did not explicitly interpret the cartilaginous element in question as a caudal skeleton, perhaps due to the absence of an ontogenetic series of the species. In so far as they commented on the presence of a small cartilage rather than an ossification at the rear of the vertebral column, it appears, based on our examination of a broad size range of specimens, that their observations were likely made on a late larva or early juvenile. No subsequent analysis delved into the question of the presence versus absence of a true caudal fin ( = hypural complex plus caudal-fin rays) in *Electrophorus*.

**Figure 2:**
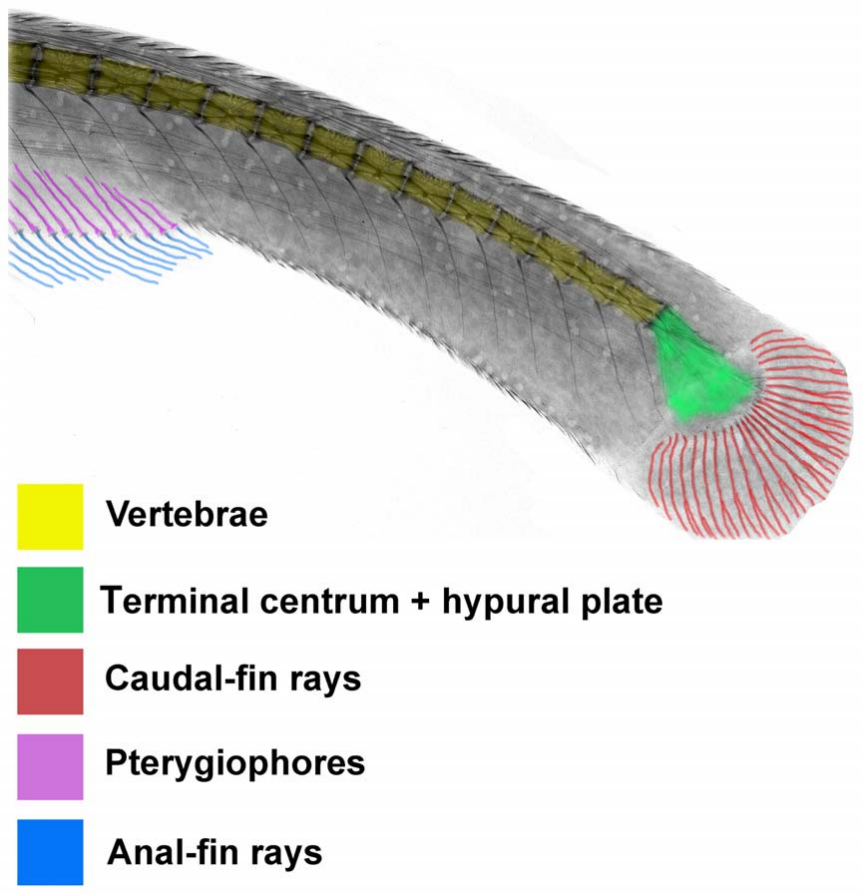
Radiographic image of caudal fin in *Platyurosternachus macrostoma,* INHS 28720, 250 mm TL. Color coding indicates components of caudal-fin skeleton, caudal-fin rays and posterior portion of anal fin.

Examination of a broad ontogenetic series of specimens of *Electrophorus* proved informative as to this question. Presence of a ventral embryological fin fold in individuals of *Electrophorus* shorter than approximately 85 mm TL gives a false first impression of a continuous anal-caudal fin during the early stages of the development in the genus. In actuality the anal-fin rays terminate well anterior to the posterior limit of the fin fold in specimens of less than this length. Larvae of *Electrophorus* of approximately 19 mm TL have anal-fin rays as evidenced by Alcian blue staining plus non-staining rays apparent in transmitted light limited to the anterior one-half of the fin fold that extends the length of the tail. Specimens at that size possess a cartilage body at the posterior end of the tail as evidenced in transmitted light without, however, any obvious associated caudal-fin rays. Conversely, fin rays are apparent at the posterior end of the tail in a circa 26 mm TL whole specimen, but with the retention of a distinct gap along the ventral margin of the tail between the posterior most apparent anal-fin ray and the ventral most caudal-fin ray. This condition is comparable to that found in adults of all species of the Apteronotidae (see Fig. 2). By approximately 60 mm TL, anal-fin rays are apparent along circa 95% of the length of the tail, but the posterior most anal-fin ray remains distinctly separate from the horizontally elongate plate-like cartilaginous mass and associated caudal-fin rays at the terminus of the vertebral column.

At 295 mm TL, the anal and caudal fins are now confluent, with the posterior most anal-fin ray as evidenced by its association with a proximal pterygiophore now situated immediately proximate to the ventral most caudal-fin ray that attaches to the hypural complex. Internally the caudal fin at this size is supported by a horizontally-elongate cartilage running ventral to the terminal portion of the notochord. The caudal fin is continued dorsally beyond the arrangment in smaller specimens by a variable number of dorsal procurrent rays within the fin fold in that region. This overall arrangement in *Electrophorus* is reminiscent of that shown for larvae of *Apteronotus leptorhynchus* by Meunier and Kirshbaum (Fig. 6 in [27]). The major difference is that in *A. leptorhynchus* all caudal-fin rays articulate solely with the posterior cartilage whereas in *Electrophorus* the dorsal caudal-fin rays attach to the ossifying notochord.

**Figure 3:**
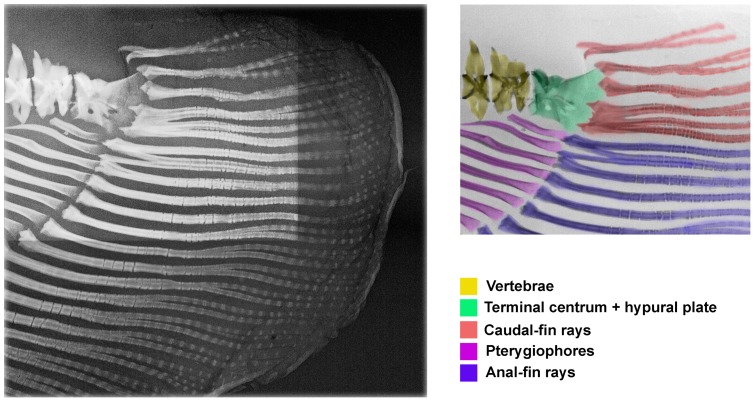
Radiographic image of caudal-fin region in *Electrophorus electricus,* USNM 225669, 500 mm TL. Color coding indicates components of caudal-fin skeleton, caudal-fin rays and posterior portion of anal fin.

**Figure 4:**
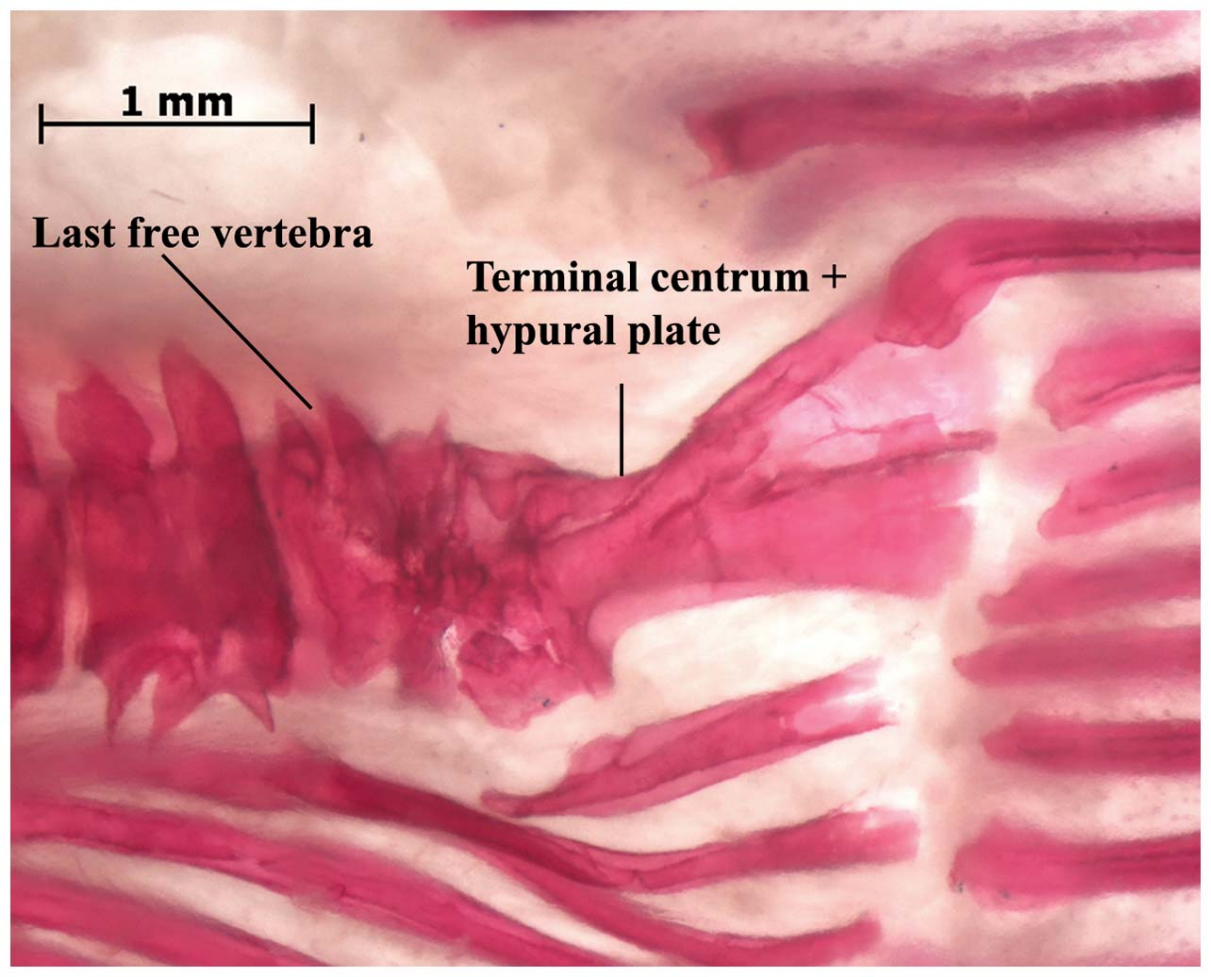
Photographic image of cleared and stained caudal-fin region in *Electrophorus electricus*, MPEG 5370, 460 mm TL.

Examination of the posterior portion of the tail in multiple samples of larger juveniles through adults of *Electrophorus* up to 1500 mm TL revealed a prominent, well ossified complex at the posterior terminus of the vertebral column (Figs. 3, 4). Two distinct components contribute to this ossification. Anteriorly, a forward facing terminal centrum contacts the posterior most independent centrum of the vertebral column via a broad articular surface comparable to those at the interfaces of the other posterior vertebrae of the vertebral column. This terminal centrum in *Electrophorus* seamlessly conjoins posteriorly with a plate-like, posteriorly vertically expanding ossification. The posterior margin of the plate-like ossification serves as the area of attachment for five to 10 caudal-fin rays; the ventral most of which adjoins the posterior most ray of the elongate anal fin. In addition to the caudal-fin rays, some specimens of *Electrophorus* possess one to three dorsal procurrent rays.

**Figure 5:**
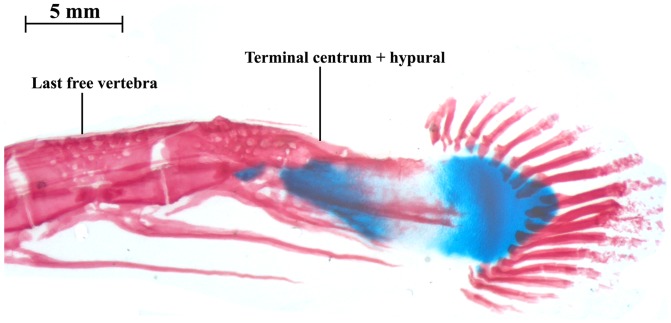
Photographic image of cleared and double stained caudal-fin region in *Apteronotus rostratus*, MBUCV 10926, 115 mm TL.

Overall morphology of the complex formed by the terminal centrum and the posterior plate of *Electrophorus* is comparable to the hypural complex at the rear of the vertebral column in most species of the Apteronotidae (Fig. 5); the one clade within the Gymnotiformes long considered to uniquely bear a true caudal fin (Figs. 2, 5; Figs. 4–5A in [27], Fig. 471 in [28], Fig. 23E in [29], Fig. 5A in [30], Fig. 1B in [31], Fig. 17A in [32]). One notable difference between the hypural complexes of *Electrophorus* and the Apteronotidae is the greater degree of ossification of that complex in midsized through adult individuals of *Electrophorus* relative to the hypural complex in most of the members of the Apteronotidae. The varying levels of ossification between these taxa may reflect the different body sizes in these taxa. *Electrophorus* is larger, sometimes significantly so (20 times), than all members of the Apteronotidae and the hypural complex of *Electrophorus* remains incompletely ossified to at least circa 300 mm TL.

Uncertainty remains about what contributes to the terminal centrum and hypural plate in *Electrophorus* and the Apteronotidae due to the reduced nature of the elements in these taxa versus the condition in other lineages in the Otophysi; for example basal members of the Siluriformes, the sister group to the Gymnotiformes. A parhypural plus six separate hypurals [33], [34] are present in *Olivaichthys viedmensis* ( = *Diplomystes papilosus* in Lundberg and Baskin [33]), a proposed basal member of the Diplomystidae [35], that, in turn, has been hypothesized in morphological analyses to be the sister group to the remainder of the Siluriformes. Siluriforms, however, demonstrate a tendency towards the fusion and/or reduction and loss of elements in the caudal skeleton in more derived taxa [34] resulting in a single bony caudal complex in some catfishes (e.g., *Chaca,* Fig. 3 in [33]; *Plotosus*, Fig. 53 in [36]) that is reminiscent of the caudal complex in *Electrophorus*.

Fink & Fink [29] proposed that the caudal plate in the apteronotid genus *Platyurosternarchus* (cited therein as *Sternarchorhamphus*) was composed of the compound centrum (the terminal centrum of Hilton et al. [32]), hypurals, parhypural and accessory hemal spine. Our examination of a broader series of specimens of the genus failed to reveal these elements as discrete ossifications during ontogeny in either species of *Platyurosternarchus*. These surveys revealed a notable degree of intraspecific variation within *P. crypticus* and *P. macrostoma* in the elaborations of the ventral portion of the terminal vertebral centrum-hypural plate complex. The accessory hemal spines of Fink & Fink [29] are most often absent (Fig. 2) and when present vary in number, form, and position and are, thus, questionably homologous with haemal spines. Analysis of examined specimens of a broad size range of *Electrophorus* proved similarly uninformative as to which elements of the typically more complex hypural system elsewhere in the Ostariophysi contribute to the posterior hypural ossification in the genus.

Elements of the reduced caudal skeleton in the Apteronotidae have been identified by several alternative terminologies. Monod [28] termed the structure an “urophore complexe.” Meunier & Kirschbaum [27], in turn, applied the name “hypuro-opisthural” to the complex. In the most recent analysis, Hilton et al. [32] found that *Orthosternarchus* has what they identified a terminal vertebral centrum (the “tv” of that study) followed posteriorly by a hypural plate (the “hp” of that study); a form of the caudal-fin skeleton comparable with that present in adults of *Electrophorus* other than for two features. The hypural plate in *Orthosternarchus* is cartilaginous and disjunct from the terminal centrum whereas in adult specimens of *Electrophorus* the hypural plate and terminal centum are both ossified and broadly conjoined (Figs. 3, 4). Nonetheless, the basic pattern of these two caudal elements is common to, and indicative of the equivalence of, the components in *Electrophorus* and *Orthosternarchus* and we apply the Hilton et al. [32] terminology for the apteronotid caudal skeleton to *Electrophorus*.

**Figure 6:**
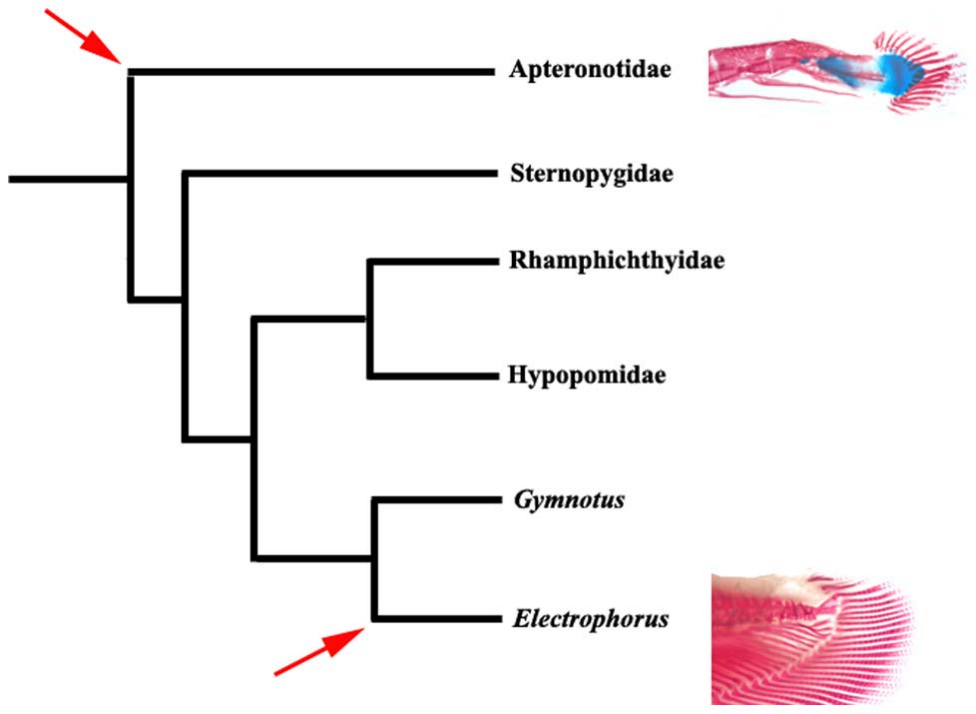
Phylogenetic relationships in Gymnotiformes based on [23], [24], [45] illustrating most parsimonious hypothesis for origins of caudal skeleton. *Electrophorus + Gymnotus  = *Gymnotidae; arrows indicate presence of caudal-fin complex in Apteronotidae and *Electrophorus.*

### The Presence of a Caudal Skeleton in *Electrophorus* and its Evolutionary Implications

Elongate bodies with associated lengthening of the anal fin characterize various taxa in the Ostariophysi; however, conjoined anal and caudal fins are restricted within the superorder to a few genera of the Gymnotiformes and Siluriformes. Analysis reveals that continuous anal and caudal fins in the Ostariophysi derive from two alternative elaborations of the separate anal and caudal fins general across the superorder: 1) a joining of the two fins at least, in part, as a result of the increase in the number of ventral procurrent rays with a consequent anterior extension of the caudal fin towards the anal fin; versus 2) the posterior extension of the anal fin to contact an unelaborated caudal fin (i.e., without an increase in the number of ventral procurrent rays). The anteroventral most ray of the caudal fin serves as an appropriate landmark for the anterior limit of that fin versus the conjoined anal fin. This ray is readily distinguished from the terminal anal-fin ray via the lack of the associated proximal pterygiophore characteristic of anal-fin rays. Additionally, the anteroventral ray of the caudal fin is most often associated with the hypural plate (the tv+hp complex of the Apteronotidae [e.g., *Platyurosternarchus* and *Apteronotus,* Figs. 2, 5]; *Electrophorus* [Figs. 3, 4]), whether the hp is ossified or partially cartilaginous.

The first of the two forms of anal-caudal fin continuity is the consequence of the caudal fin extending anteriorly to varying degrees along the ventral margin of the body to meet a posteriorly extended anal fin. This state can be recognized by the presence of multiple ventral procurrent caudal-fin rays lacking associated proximal pterygiophores posterior of the terminal anal-fin ray as demarked by the posterior most proximal anal-fin pterygiophore. Within the Siluriformes, this morphology was observed in the Neotropical genus *Phreatobius* which has 11 to 26 ventral procurrent rays [37]–[39] and the African genus *Gymnallabes* in which there are at least five ventral procurrent rays extending forward to meet an posteriorly extended anal fin (Fig. 5 in [40]).

The second, and non-homologous, mode of continuity between the anal and caudal fins is achieved via the posterior extension of the anal fin to contact a non-anteriorly lengthened caudal fin (i.e., without multiple ventral procurrent caudal-fin rays). This condition is characterized by the immediate proximity of the posterior most anal-fin ray as evidenced by an associated proximal pterygiophore with the ventral most caudal-fin ray; the condition found in *Electrophorus.* As detailed above, the anal fin in *Electrophorus* progressively expands posteriorly during ontogeny until the posterior margin of that fin reaches and conjoins the anteroventral margin of the caudal fin thereby yielding a continuous anal-caudal fin complex (Fig. 3). Elsewhere in the Ostariophysi, an anal fin confluent with the caudal fin as a consequence of the posterior extension of the anal fin to conjoin a non-anteriorly lengthened caudal fin is also known to occur in the Plotosidae, a family of marine and freshwaters catfishes of the Indo-Pacific region (Fig. 53 in [36]). The Plotosidae is well embedded within the Siluriformes based on both morphological [41] and molecular data [42], and the monophyly of the Gymnotiformes is, in turn, supported by multiple synapomorphies [1]. Thus, the conjunction of the anal and caudal fins via the posterior elongation of the anal fin in *Electrophorus* is clearly homoplastic relative to the similar condition in the Plotosidae. Given that continuity between the anal and caudal fins as a consequence of the posterior expansion of the anal fin to contact the ventral-most caudal-fin ray is unique to *Electrophorus* in the Gymnotiformes, that condition serves as an additional autapomorphy for the genus.

In so far as it had been assumed that the caudal fin was absent in *Electrophorus*, information on the number of caudal-fin rays for that genus was not included in prior phylogenetic analyses. Within the Apteronotidae, the only other group in the order with a caudal fin, the number of rays ranges from five to 30 with the basal clades, such as that formed by *Orthosternarchus* plus *Sternarchorhamphus*, possessing five to nine rays and the other genera in the family 10 to 30 rays (e.g., *Platyurosternarchus*, Fig. 2; *Apteronotus*, Fig. 5). The four to 10 caudal-fin rays in *Electrophorus* (Fig. 3), thus, parallel the count for hypothesized basal apteronotids.

According to prior literature, a true caudal fin formed by a terminal vertebral centrum (tv) and hypural plate (hp) is restricted in the Gymnotiformes to members of the Apteronotidae, the most speciose family in the order [1], [17], [18], [22]–[25], [27], [29], [43], [44]. The presence of an apteronotid form of tv+hp complex and caudal fin in *Electrophorus* contra the previous assumption of the lack of those systems in that genus may impact previous hypotheses of phylogenetic relationships within the Gymnotiformes. Indeed in isolation, this discovery raises the question of whether the absence of a caudal fin in all taxa of the Gymnotiformes other than the Apteronotidae and *Electrophorus* is a potential synapomorphy for a clade composed of all members of the order lacking the fin. Examination of the impact of the discovery of a caudal fin in *Electrophorus* on prior hypothesis would necessitate not only the inclusion of information concerning the presence of a caudal-fin and caudal-fin rays in the genus, but also the incorporation of the extensive data from recently published phylogenetic analyses of various genera within the order, e.g., [5]–[7]. That undertaking lies beyond the purpose of this study. Nonetheless, there are two primary hypotheses of phylogenetic relationships among Gymnotiformes reiterated in the last two decades that serve as a framework for an interpretation of the presence of the caudal skeleton in *Electrophorus*.

The most significant divergence between these involves the taxa judged to be the sister group to all other members of the order. The first of these hypotheses – that the Apteronotidae (with a caudal and caudal-fin rays) is the sister group to all other families in the Gymnotiformes was advanced based on morphological [23], [24] and molecular [45] data (Fig. 6). Under that scenario the presence of a caudal fin in the Apteronotidae is most parsimoniously hypothesized to represent the retention of the plesiomorphic condition common to all members of the Siluriformes, the sister group to the Gymnotiformes. Both of the morphological analyses [23], [24] have *Electrophorus* separated from the Apteronotidae within the Gymnotiformes by three nodes. Given the phylogenetic distance between the Apteronotidae and *Electrophorus,* the most parsimonious explanation for the distribution of a caudal fin in the two lineages involves retention of the caudal fin in the basal Apteronotidae, the loss of the fin in the ancestor of the remainder of the order, and a reacquisition of the fin in *Electrophorus*. This involves fewer evolutionary steps than the perhaps intuitively more appealing hypothesis of multiple loses of the fin in the Sternopygidae, the ancestor of the Hypopomidae plus Rhamphichthyidae, plus *Gymnotus* in the Gymnotidae (Fig. 6). The molecular study [45] includes fewer taxa, but again the hypothesis of an independent caudal fin acquisition in *Electrophorus* is the most parsimonious within the context of the phylogeny.

**Figure 7:**
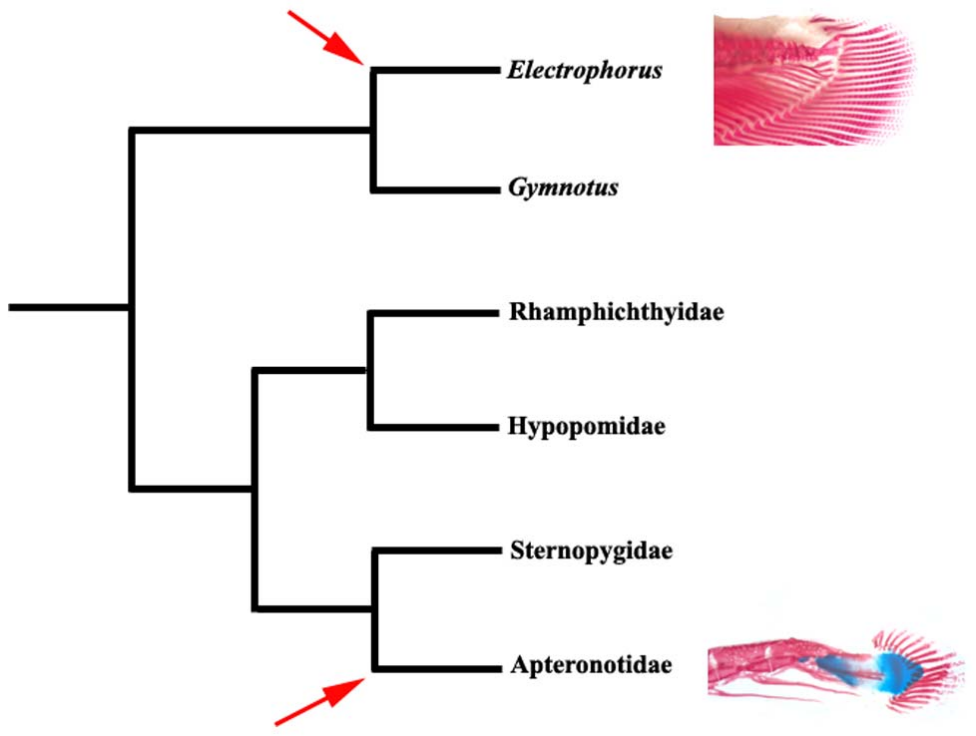
Phylogenetic relationships in Gymnotiformes based on [1], [17], [25] illustrating most parsimonious hypothesis for origins of caudal skeleton. *Electrophorus + Gymnotus  = *Gymnotidae; arrows indicate presence of caudal fin complex in Apteronotidae and *Electrophorus.*

The second major phylogenetic hypothesis of relationships for the Gymnotiformes, this based on morphological data, alternatively has the Gymnotidae (*Electrophorus* plus *Gymnotus*) as the sister clade to the remainder of the order [1], [17], [25] (Fig. 7). Under that scenario the Apteronotidae is a crown group within the Gymnotiformes separated by four nodes from *Electrophorus.* Within this phylogenetic scheme, the presence of a caudal fin in those taxa again optimizes as separate events, with two alternative equally parsimonious explanations. Under one, the presence of the caudal fin in the Apteronotidae and *Electrophorus* represents separate acquisitions post the presumed loss of the complex in the ancestor of the Gymnotiformes. The second scheme involves the loss of the fin in *Gymnotus* (the sister group to *Electrophorus*) in the Gymnotidae and in the ancestor of the Rhamphichthyidae, Hypopomidae, Sternopygidae and Apteronotidae and the reacquistion of the fin in the Apteronotidae (Fig. 7).

Under all of these phylogenetic hypotheses, the distribution of a caudal complex and fin within the Gymnotiformes would potentially involve the retention of a plesiomorphic condition (presence of the tv+hp and caudal fin) or acquisition of the fin in a clade sister to the remainder of the order and a secondary presence of the caudal complex in another lineage. The alternatives mirror each other with the presence of a true caudal fin in *Electrophorus* being the secondary presence under the phylogenetic hypothesis of the Apteronotidae as sister to a clade formed by other families (Fig. 6) and the occurrence of the fin in the Apteronotidae being a secondary presence under the assumption of the Gymnotidae (including *Electrophorus*) being the sister of the remainder of the Gymnotiformes (Fig. 7).

### Functional Considerations

Absence of the caudal fin is common to many components of the Gymnotiformes, but overall is limited to relatively few groups within the Teleostei; a not unexpected situation in so far as the caudal fin provides the majority, or a significant portion, of the propulsive force to the fish along with contributing to steering functions. A universal lack of the pelvic fin across Neotropical electric fishes in addition to the general absence of the caudal fin is also noteworthy. Although the pelvic fins are not a major factor in propulsion across fishes, they contribute to fine movement control. Offsetting the loss of these two fins across the Gymnotiformes is a dramatic lengthening of the anal fin and increased fine motor control of propulsive movements within the fin. Depending on the taxon, the gymnotiform anal fin commences anteriorly within the region between the vertical through the orbit to the posterior limit of the abdominal cavity and continues caudally to varying positions along, or at the end of, the tail (see figures in [25]; Figs. 1–3). Reflecting the pronounced elongation of the anal fin are anal-fin ray counts of circa 100–400 across the order; numbers that are dramatically higher than in most other taxa in the Ostariophysi [1], [25].

Sinusoidal movements along this elongate anal fin among species of the Gymnotiformes provide the primary propulsive mechanism for the distinctive anterior and posterior movements of these fishes and in conjunction with the pectoral fin, critical fine scale control of such movements [46]. Fine control of posterior motion is a necessity for effective foraging behavior among gymnotiforms, with movement of the rigid body anteriorly and posteriorly prerequisite for scanning potential prey items via the electroreceptive array on their skin [47], [48]. Dependence on the anal fin for propulsion in conjunction with the necessity of a straight alignment of the body for electroreceptive functioning diminished the propulsive importance of the caudal fin. This reduced or obviated the need for a substantial caudal fin; a system which is absent across the Gymnotiformes with the exception of the Apteronotidae and *Electrophorus*. Strikingly similar absences of the caudal and pelvic fins occur in the African electrogenic genus *Gymnarchus* (Osteoglossiformes). *Gymnarchus* also swims with a largely rigid body and propels itself via sinusoidal movements along an elongate median fin; the propulsive fin in that genus being, however, the dorsal rather than anal fin yielding an amiiform swimming mode [46].

The Gymnotidae is unique within the Gymnotiformes in demonstrating intrafamilial variation in the presence versus absence of the caudal fin, with the fin present in *Electrophorus* versus absent in its sister group, *Gymnotus.* A potential functional difference underlying this variation may be the rigid body posture in life of species of *Gymnotus* with sinusoidal movements along the anal fin generating the primary propulsive force [46], [48]. Conversely, *Electrophorus* demonstrates two alternative swimming modes. The first of these is the straight alignment of the body during obligate gulping of air and in the detection, location and shocking of prey items. This is the body orientation general across the Gymnotiformes, e.g., [46], [48]. *Electrophorus* is additionally able to use sinusoidal or anguilliform movements along the length of the entire body to supplement the waves of movements along the anal fin during capture of prey and rapid forward motion. During this swimming mode, the posterior portion of the body undergoes pronounced side-to-side movements; a situation in which a caudal fin would increase the anterior propulsive force and thereby be functionally advantageous as is the case with other groups of fishes using anguilliform swimming modes. Taxa of the Apteronotidae which also have caudal fins lack, however, anal-caudal fin conjunction and is there no indication of alternative swimming modes in the family.

## Supporting Information

List S1 **List of specimens of** ***Electrophorus*** **and outgroups examined in this study.**
(DOC)
