# Supplementary material for: The Untold Story of the Caudal Skeleton in the Electric Eel (Ostariophysi: Gymnotiformes: Electrophorus)
Source: PLoS One. 2013 Jul 24;8(7):e68719. doi: 10.1371/journal.pone.0068719 (PMC3722192; doi:10.1371/journal.pone.0068719)
Supplement: List S1 — List of specimens of Electrophorus and outgroups examined in this study. (DOC) [file pone.0068719.s001.doc]

**S1.** List of material examined for *Electrophorus electricus* and outgroups. Material is ordered alphabetically by species, country and, collection institution and numerically by collection lot number. Geographical coordinates are provided when originally associated with specimens. Body size is reported in mm of TL. Radiographed specimens are indicated by r and cleared and stained specimens as c&s.

*Electrophorus electricus* (Linnaeus), Brazil. MPEG 2973, 3(2 c&s), 67.0–74.0; MPEG 2974, 3(1 c&s), 18.2–23.8; MPEG 2975, 4(2 c&s), 32.0–33.5; MPEG 5370, 2(r), 432–460; Pará, Ilha do Marajó, Rio Goaipi, Cachoeira do Arari. MPEG 2799, 2(1 c&s), 201–213; MPEG 2976, 3(2 c&s), 17.0–18.0; MPEG 2978, 9 (2 c&s), 12.5–13.6; MPEG 3918, 15(1 c&s), 22.0–23.0; MPEG 4019, 11(2 c&s), 11.3–12.3; Pará, Ilha do Marajó, Rio Goaipi, Taperebá, Fazenda Santa Maria, Cachoeira do Arari. USNM 196634, 8(r), 730–1400; Rio Amazonas on Stegeaman ranch, Maraga Detta. USNM 198961, 1(r), 800; USNM 399730, 1(r), 380; Mato Grosso, Rio Batovi, upper Rio Xingu, Waura Indian Village. Ecuador. FMNH 94904, 1(r), 215; Río Conambo, Río Tigre. KU 13803, 1(r), 1110; KU 13804, 1(r), 210; Napo, stream tributary of Río Conejo at Santa Cecilia. Guyana. AMNH 72973, 1(r), 600; Essequibo River basin, sandbar on north bank of Cuyuni River, just upstream of Caowry creek, Mazaruni-Potaro District. AUM 44613, 1(r), 310; Takutu River, beach near Lethem. USNM 403765, 1(r), 232; Cuyuni-Mazaruni. Peru. ANSP 22010, 1(r), 410; Pebas, Río Amazonas. INHS 43681, 1(r), 370; Ulpa Caño and Moema Caño. NRM 27643, 1(r), 375; Río Samiria, quebradita tributary to Quebrada Santa Elena. NRM 27649, 2(r), 340–355; Río Yavari, small cocha on left bank of Río Yaquerena about 2 hours upstream of Colonia Angamos. UF 116585, 3(1 c&s; 2R), 295–450; Río Amazonas, white water cocha near Iquitos. UF 129822, 1(r), 320; Río Pacaya in Reserva Nacional Pacaya Samiria. Suriname. USNM 225266, 1(r), 148; Kamp Creek 100 m north of turnoff to Kamp Geology; USNM 225576, 1(r), 1000; Kapoeri Creek, approximately 4 km N from intersection with Corantijn River. USNM 225668, 1(r), 550; Corantijn River, near Tiger waterfall, 04°00’00”N 058°02’00”W; USNM 225669, 1(r), 500; Corantijn River, near to Amopoto, 03°33’00”N 057°40’00”W; USNM 225670, 1(r), 520; Corantijn River, 03°27’00”N 057°37’00”W; USNM 225671, 1(r), 550; Corantijn River, pool in front of Camp Hydro, 03°42’00”N 057°58’00’’W. Venezuela. ANSP 141593, 7(r), 350–420; Río Orinoco, isolated lagoon 200 yards N of Jabillal. ANSP 192860, 1(r), 100; Río Bacural, Río Arauca, bridge between San Juan de Payare and Paso Arauca. AUM 43957, 1(r), 630; Río Orinoco, 33.9 km W of La Esmeralda, Puento Piaroa. USNM 228883, 2(r), 1400–1555; Delta Amacuro, Río Orinoco, small caño and marsh on S shore, 83 nautical miles upstream from sea buoy.

**Outgroups**

*Apteronotus albifrons* (Linnaeus), Venezuela. USNM 260256, 1(c&s), 133; Guarico, Río Caracol where crossed by bridge on ranch.

*Apteronotus cuchillo* Schultz, Venezuela. MBUCV 7554, 1(c&s), 145; Zulia, Río Apon, approximately 35 km S of Villa del Rosaria, Lago Maracaibo basin. MCZ 52010, 1(c&s), 180; Venezuela, Zulia, Río Motatán at bridge, 22 km N of Motatán.

*Apteronotus rostratus* (Meek & Hildebrand), Panamá. MBUCV 10926, 2(c&s), 110–115; Río Pierre, tributary of Río Tuyra.

*Orthosternarchus tamandua* (Boulenger), USNM 373017, 1(c&s), 240; Brazil, Amazonas, Rio Solimões.

*Platyurosternarchus crypticus* de Santana & Vari, Guyana. MCZ 46889, 2 (c&s), approximately 90–100; MCZ 98431, 1 (c&s), 175; Rupununi, Moco-Moco Creek, upper Takutu River (Rio Branco basin), N slope of base of Kanuku Mountains.

*Platyurosternarchus macrostoma* (Günther), Colombia. MBUCV 12770, 1(c&s), 207; Meta, Río Meta, Quebrada Ventuorsa above road between La Balsa and Puerto Lopez. Venezuela. FMNH 100730, 2(c&s), 156–213; Barinas, Caño Socopo, Río Suripa basin. INHS 28720, 1, 250(r); Río Santo Domingo in Torunos.

*Sternarchorhamphus muelleri* (Steindachner), Venezuela. USNM 228807, 6 (c&s), 162–326; Anzoátegui, La Providencia, N side of Isla Fajardo, opposite Palua.

*Sternarchorhynchus mormyrus* (Steindachner)*,* Brazil. USNM 306843, 1(c&s), 113; Amazonas, Rio Amazonas, 28.5 km below Manaus.
